# COVID-19–Related Racism and Mental Health Among Asian Americans: Integrative Review

**DOI:** 10.2196/63769

**Published:** 2025-04-02

**Authors:** Tania Von Visger, Amy Lyons, Yanjun Zhou, Kayla Wardlaw, Eunhee Park, Yu-Ping Chang

**Affiliations:** 1 School of Nursing, State University of New York at Buffalo, 3435 Main St, Wende Hall Suite #201F, Buffalo, NY, 14214, United States, 1 7168292201

**Keywords:** racism, anti-Asian sentiment, integrative review, psychological distress, mental health, review, Asian American, Asian, wellness, psychological, distress, COVID-19, pandemic, cross-sectional survey, survey, depression, anxiety

## Abstract

**Background:**

Racism against Asian Americans escalated during the COVID-19 pandemic. About 31%‐91% of Asian American adults and children reported experiencing various types of racism during the pandemic. According to the Federal Bureau of Investigation hate crime statistics, anti-Asian hate crime incidents increased from 158 in 2019 to 279 in 2020 and 746 in 2021. In 2022, the incidents decreased to 499, corresponding to the downward trend of the pandemic. The degree of impact racism has on mental health and wellness among Asian Americans requires investigation, specifically during the COVID-19 pandemic.

**Objective:**

We aim to describe racism-related mental health problems experienced by Asian Americans living in the United States and propose implementation strategies for mitigating their consequences.

**Methods:**

We conducted an integrative review of peer-reviewed publications in English reporting anti-Asian sentiments and racism’s impacts on mental health among Asian Americans in the United States.

**Results:**

The 29 eligible articles report on studies that utilized cross-sectional survey designs with various sample sizes. Racism is directly correlated with the prevalence of depression and anxiety experienced by victims of racist acts. The prevalence of in-person direct racism (racist expression aimed directly at the victim) is lower than in-person indirect racism (racist expression aimed at the ethnic group the victim belongs to). During the COVID-19 pandemic, the incidence of explicit online racism was lower than online indirect racism.

**Conclusions:**

COVID-19–related racism exacerbated preexisting racism, contributing to worse depression and anxiety among Asian Americans. To address this issue, we propose 2 main approaches: increase public awareness and education about recognizable racist sentiments/acts and systematized reporting of racially motivated crimes to guide political action. At an individual level, culturally responsive, trauma-informed interventions promoting cultural support and cohesion for various Asian American groups will foster this empowerment. These proposed actions will help alleviate racism by reducing stereotypes, empowering victims, and chipping away at the systemic racism structure.

## Introduction

### Background

In June 2021, the National Commission to Address Racism in Nursing defined racism as “assaults on the human spirit in the form of actions, biases, prejudices, and an ideology of superiority based on race that persistently causes moral suffering and physical harm to individuals and perpetuates systemic injustices and inequities” [1]. From this broad perspective, there are ranges of defining characteristics depending on how the perpetrators commit the assault and whether the assault is overt, as well as the detrimental outcomes (physical or emotional) of the assaults. More importantly, displays and the impacts of racism vary in form and intensity and can have long-lasting deleterious effects on the social environment. Because racism broadly exists at 3 connected levels, multiple approaches to addressing the issue are necessary. These levels include institutional racism (policies and practices that allow inequity), cultural racism (ideology of inferiority of particular groups), and discrimination (individual level) [2].

Observable and objective consequences of racism, such as physical harm, occur less often than other forms of racism; however, other forms may be more challenging to identify, and psychosocial suffering may be hidden and rarely discussed [3,4]. The complexity of these multiple dimensions and domains of racism (eg, displays of racism [beliefs, words, or actions]; impacts of racism [emotional or physical harms]; and reach of racism [individual, family, community, or society]) presents challenges for defining and measuring constructs of racism and the true psychological impacts on victims. The relationships between racism, perpetrators of racism, and victims of racism do not exist in a vacuum; they are shaped by many cultural racism-related social factors throughout US history [5,6]. The stereotypes baked into the human psyche turn into norms and environmental structures of institutional or systemic racism [7-9]. Systemic racism becomes part of our society and social structure, perpetuating systemic injustices and inequities embedded and reinforced in laws and regulations and unconsciously in biases and prejudices [10].

Verbal or written expressions of racist attitudes toward a specific racial-ethnic group are considered “hate speech.” Regardless of the delivery method of racist expression (direct vs indirect) and the intensity of the racist expression (subtle microaggressions to overtly racist remarks), individual-level racism (such as hate speech) and discrimination are associated with poor mental health and a higher risk of psychiatric disorders across minority groups [11]. Racism has also been shown to affect physical health through the mechanism of the physiologic stress response, as measured by chronic inflammatory markers, and it also directly links to increased depressive and anxiety symptoms [12]. In a meta-analysis involving 293 studies, racism was associated with poor mental health (depression, anxiety, and psychiatric distress) and poor general health and physical health [13]. One study showed everyday discrimination is associated with higher odds of developing psychiatric experiences (odds ratio [OR] 4.59) and lifetime psychotic experiences (OR 4.27) in a large sample that included Latino, Asian, African American, and Caribbean Black adults [12]. The association between racism and mental health was stronger among Asian Americans than among Blacks [13]. Everyday discrimination experiences among Asian Americans increased the likelihood of being diagnosed with depression (OR 1.72) and anxiety (OR 2.24) disorders within the past 12 months while controlling for confounding variables such as poverty level, acculturation, physical health, family cohesion, and social desirability [14]. Among studies that explore Asian American subgroups, racism is associated with poor mental health in college students, East Asian adults, and South Asian adults [15-17]. Regardless of the range of mental health severity impacts, racism experiences among minoritized groups yield consistent results of harmful psychological and physical health deterioration.

### Displays of Individual-Level Racism

Expression or displays of racism can vary in subtlety, from microaggressions to more deadly acts of physical assaults. Racist words can be directly aimed at an individual, which is called direct racism, or at the minority group identified by the individuals, termed indirect or vicarious racism [18]. Those who experience repeated racism may harbor a constant state of fear and heightened awareness called racial discrimination vigilance [11]. The developmental theory of embodiment emphasizes the strong connection between the social environment and how people come to understand the world around them, such that the social structure of domination and privilege can lead to the embodiment of health inequities [19]. This internalization of unjust social premises is an antecedent to conscious and unconscious vigilance, leading to poorer mental and physical health [19]. Due to the COVID-19 pandemic lockdown, more racist attacks were perpetuated online, although face-to-face or in-person racist acts continued to rise as well [18,20-23]. The increased use of social media during and immediately after the lockdown contributed to an increase in online anti-Asian sentiments, as these platforms are venues where people tend to be uninhibited [24]. An analysis of more than 1 million social media hashtags distinguishing the degree of anti-Asian sentiment association with word choice revealed that 50.4% (392,037/777,852) of #ChineseVirus contained anti-Asian sentiments compared to 19.7% (of 495,289) of #COVID-19 [25]. The use of these terms increased dramatically from March 9 to 23, 2020; the degree of increase was statistically different between #ChineseVirus and #COVID-19 [25]. Regardless of the method or intensity of anti-Asian racist expression, data indicate that all types of racism negatively impact Asian American individuals’ mental health and well-being. This impact is especially pronounced when racism is perpetrated through social media platforms [25]. Throughout this article, we use “anti-Asian sentiment” to represent the central concept encompassing this broader display of racism, whether subtle or overt.

### Racism Experienced by Asian Americans in the Context of the COVID-19 Pandemic

In the last weeks of 2019, COVID-19 emerged in Wuhan, China, eventually leading to a worldwide lockdown in March 2020. COVID-19 is related to an earlier viral strain that caused a smaller-scale pandemic in November 2002 with clinical presentation of severe acute respiratory syndrome. Both phenomena originated in China, fueling the spread of misinformation and anti-Asian sentiments. Studies indicate that anti-Asian sentiments (racist sentiments, including microaggression and unconscious biases) increased significantly during the COVID-19 pandemic [18,20,21,24-27]. Like other racial minorities living in the United States, racism against Asian Americans is not a recent phenomenon; it persists throughout American history, as documented in the Yellow Peril, Japanese internment, and the perpetuation of the model minority sentiment [5,6]. The intensity and wide range of racist displays against Asian Americans, including social media posts [24,28], discriminatory behaviors, and hate incidents, dramatically increased during the COVID-19 pandemic [20,26]. Systematic reviews about Asian American hate or Asian American hate combined with mental health during the COVID-19 pandemic are limited. We are mindful that an accurate assessment of the impact of the COVID-19 pandemic is limited by incomplete and inaccurate reporting of physical and mental health outcomes [29].

We conducted an integrative review to explore mental health problems experienced by Asian Americans during the COVID-19 pandemic. The purpose of this review is to understand how anti-Asian sentiments (prejudice, hatred, or racism) impact mental health (anxiety, depression) among Asian Americans (Asian immigrants and Asian Americans) living in the United States during the COVID-19 pandemic. Guided by the integrative review procedures, we focused our research investigation on the PICO question, “How do anti-Asian sentiments (prejudice, hatred, or racism) impact mental health (anxiety, depression) among Asian Americans (Asian immigrants and Asian Americans) living in the United States during the COVID-19?”

## Methods

### Study Design

We followed the integrative review methodology described by Whittemore and Knafl (2005) [30], which includes five general steps: (1) defining the target population and problem, (2) literature search, (3) data evaluation, (4) data analysis, and (5) data presentation. The Preferred Reporting Items for Systematic Reviews and Meta-Analyses (PRISMA) were used to guide the reporting of this review.

### Search Strategies

From June 2021 to November 2023, the authors, including a librarian with expertise in conducting systematic reviews, searched multiple electronic databases. Searched databases were Embase, PubMed, CINAHL, Cochrane, PsycINFO, and Web of Science. We extensively searched for publications reflecting the impact of the COVID-19 pandemic lockdown officially implemented in March 2020. We included articles published between January 1, 2020, and November 31, 2023, to include the effect of COVID-19 in the initial phase, even before the national announcement of the COVID-19 lockdown mandate.

### Study Selection and Inclusion and Exclusion Criteria

We searched for peer-reviewed publications of empirical literature reporting about anti-Asian sentiments and mental health and well-being among Asian Americans in the United States during the COVID-19 pandemic. Search terms used in the study were as follows: Asian Americans AND COVID-19, AND mental health–related terms (mental health OR depression OR anxiety), AND terms related to racism (racism OR model minority OR health inequity). An initial search in PubMed and CINAHL helped to identify a complete list of key search terms that accurately described our aim of identifying relevant articles. We then applied these search terms to identify references in related databases. For inclusion in this review, studies must: (1) be published in peer-reviewed research journals; (2) focus on Asian American populations; (3) report on anti-Asian sentiment, individual-level racism, or discrimination; and (4) report on mental health outcomes. We excluded the following: (1) conference abstracts, (2) opinion or discussion reports, (3) systematic reviews, (4) research not published in English, and (5) research not conducted in the United States.

### Data Extraction

**Figure 1.**
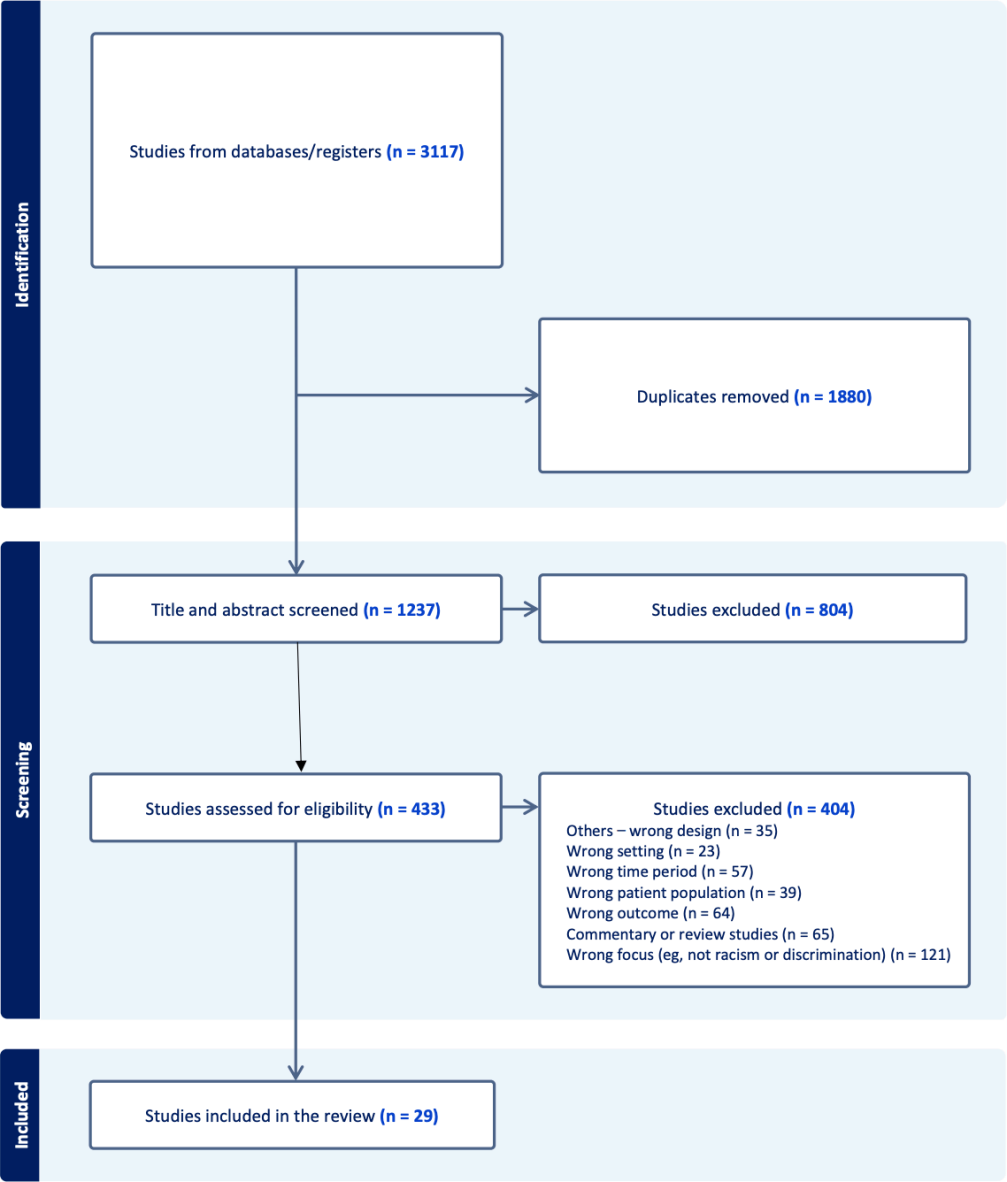
Preferred Reporting Items for Systematic Reviews and Meta-Analyses (PRISMA) flowchart: search results and study selection procedures.

Figure 1 shows a flow diagram of the study screening process. From the original 3117 citations identified, 1880 duplicates were removed, with 1237 remaining. Two researchers independently reviewed titles and abstracts and agreed to exclude 804 publications because they did not include Asian Americans, were not conducted during the COVID-19 pandemic, did not measure mental health outcomes, or were commentaries. The 433 remaining articles received a full-text review by 2 independent reviewers, with a third reviewer to resolve the disagreements. A total of 404 articles were excluded.

### Synthesis of Findings

Two authors independently reviewed 29 articles. They coded them into a table format, including details about participants’ characteristics, settings, sample size, study designs, aims, statistical methods, and main results. The primary clinical outcomes investigated and reported to be associated with racism in the articles reviewed were depression and anxiety. The same 2 reviewers compared abstracted data and then discussed the data with a third reviewer to achieve consensus.

## Results

### Quality Appraisal

We used the Joanna Briggs Institute (JBI) guidelines for appraising cross-sectional research to assess the quality of the reports (Table 1). The JBI Critical Appraisal Checklist for Cross-Sectional Studies includes “yes” or “no” confirmation of 8 criteria regarding the overall conduct of the investigations surrounding the scientific rigors about study subjects and settings, standard condition measurement, reliable and valid outcome measurements, appropriate statistical analysis, and description of confounding factors (Table 1).

**Table 1.** Quality assessment of studies included in the review (n=29).

	Were the criteria for inclusion in the sample clearly defined?	Were the study subjects and the setting described in detail?	Was the exposure measured in a valid and reliable way?	Were objective, standard criteria used for measurement of the condition?	Were confounding factors identified?	Were strategies to deal with confounding factors stated?	Were the outcomes measured in a valid and reliable way?	Was appropriate statistical analysis used?
Chae et al, 2021 [11]	Y^a^	Y	Y	Y	Y	Y	Y	Y
Cheah et al, 2020 [22]	Y	Y	Y	Y	N/A^b^	N/A	Y	Y
Cheah et al, 2023 [31]	Y	Y	Y	Y	Y	Y	Y	Y
Dhanani et al, 2022 [32]	Y	Y	Y	Y	Y	Y	Y	Y
Ermis-Demirtas et al, 2022 [33]	Y	Y	Y	Y	Y	Y	Y	Y
Fanta et al, 2023 [5]	Y	Y	Y	Y	Y	Y	Y	Y
Haft and Zhou, 2021 [34]	Y	Y	Y	Y	Y	Y	Y	Y
Huynh VW et al, 2022 [35]	Y	Y	Y	Y	N^c^	N/A	Y	Y
Huynh VW et al, 2022 [35]	Y	Y	Y	Y	N	N/A	Y	Y
Huynh J et al, 2022 [36]	Y	Y	Y	Y	Y	Y	Y	Y
Ikram et al, 2023 [37]	Y	U^d^	Y	Y	N	N/A	Y	Y
Keum and Choi, 2022 [38]	Y	U	Y	Y	Y	Y	Y	Y
Layug et al, 2022 [39]	Y	Y	Y	Y	N	N/A	Y	Y
Lee et al, 2020 [10]	Y	U	Y	Y	N/A	N/A	Y	Y
Li et al, 2023 [40]	Y	Y	Y	Y	Y	Y	Y	Y
Litam and Oh, 2022 [41]	Y	U	Y	Y	Y	Y	Y	Y
Litam et al, 2022 [41]	Y	U	Y	Y	Y	Y	Y	Y
Liu et al, 2020 [42]	Y	Y	Y	Y	Y	Y	Y	Y
Liu et al, 2022 [43]	Y	Y	Y	Y	Y	Y	Y	Y
Lu et al, 2022 [44]	Y	U	Y	Y	N	N/A	Y	Y
McGarity-Palmer et al, 2023 [45]	Y	Y	Y	Y	Y	Y	Y	Y
Oh et al, 2022 [46]	Y	U	Y	Y	N	N/A	Y	Y
Oh et al, 2022 [47]	Y	U	Y	Y	Y	Y	Y	Y
Pan et al, 2020 [48]	Y	Y	Y	Y	Y	Y	Y	Y
Jun et al, 2021 [49]	Y	Y	Y	Y	Y	Y	Y	Y
Wu et al, 2021 [50]	Y	Y	Y	Y	Y	Y	Y	Y
Zhou et al, 2023 [51]	Y	Y	Y	Y	Y	Y	Y	Y

a Y: Yes.

b N/A: Not applicable.

c N: No.

d U: Unclear.

### Study Characteristics

All 29 studies, with a wide range of sample sizes (from N=64 to N=7813), utilized cross-sectional survey designs conducted during the COVID-19 pandemic starting in March 2020, assessing possible associations between individual-level racism and mental health outcomes (Table 2 and Figure 1). All 29 articles received a score above 7 using the JBI Critical Appraisal tool. Three articles provided mental health outcomes of children as direct recipients of racism, directly or indirectly, through their parents [5,23,31]. These studies measured individual-level racism, focusing on direct racism; four assessed vicarious racism, and one assessed vigilance [3,9,22,23,31,39]. Five studies distinguished between online racism and in-person racism, and one differentiated the victim experience between US-born and foreign-born Asian Americans [22,23,31,33,44,48]. Our synthesis of available data revealed that racism against Asian Americans during the COVID-19 pandemic in the United States impacted the 2 mental health outcomes of depression and anxiety.

**Table 2.** Literature review matrix summary.

Publication	Sample population	Time frame	Racism variables	Mental health outcomes	Main findings	Strengths/significance
1. Chae et al, 2021 [11]	604 Asian Americans; 844 Blacks in 5 US cities	May 21, 2020, to July 15, 2020	Vicarious racism (7-item Likert); vigilance (4-item Likert)	Depression and anxiety	Racism is associated with depression (Asian Americans: *b*=1.92; Blacks: *b*=1.72); racism is associated with anxiety (Asian Americans: *b*=2.4; Blacks: *b*=1.98); vicarious racism is associated with depression (Asian Americans: *b*=1.54; Blacks: *b*=0.90); vicarious racism is associated with anxiety (Asian Americans: *b*=1.98; Blacks: *b*=1.65)	Limited to 5 US cities
2. Cheah et al, 2020 [22]	543 Chinese Americans and their 230 children	March 14, 2020, to May 31, 2020	Online direct racism; online vicarious racism; in-person direct racism; in-person vicarious racism; health-related Sinophobia; Sinophobia in media	Psychological well-being (Ryff’s 18-item); GAD-7^a^, Beck Depression	Prevalence of racism: online racism (31.7% adults; 45.7% youths); online vicarious racism (76.8% adults; 76.5% youths); in-person racism (50.9% adults; 50.2% youths); in-person vicarious racism (88.5% adults; 91.9% youths). Psychological well-being was negatively associated with most types of racism. Anxiety and depression were positively associated with all types of racism.	Measured 6 types of racism in Chinese Americans and their children
3. Cheah et al, 2023 [31]	529 Chinese American parents and their 225 children (4‐18 years old)	2020 and 2021	Health-related Sinophobia; media Sinophobia: online direct, online vicarious, in-person direct, in-person vicarious	Psychological well-being (Ryff’s 18-item); GAD-7; Beck Depression	Dimensions of racism were more strongly associated with parent anxiety symptoms (interaction effect *b* values ranging from 0.74 to 1.76; *P* values ranging from .004 to <0.001) in 2021 than in 2020. Parent online vicarious discrimination was more strongly and positively associated with parent depressive symptoms (interaction effect *b*=1.03; *P*<.001). Racism dimensions were more strongly associated with youth anxiety symptoms (interaction effect *b* ranging from 0.92 to 1.65; *P* values ranging from .013 to <.001) in 2021 than in 2020.	Demonstrated the increased gravity of the mental health issues with time
4. Dhanani et al, 2022 [32]	Asians and Asian Americans (T1 only: n=401; T1 and T2: n=311)	April 2021	Experienced discrimination (Everyday Discrimination Scale); vicarious discrimination (adapted version of 3-item Vicarious Racism Scale)	PHQ^b^-9	Significant association with increased depression (*r*=0.31, *P*<.001), physical health symptoms (*r*=0.32, *P*<.001), and increased sleep disturbances (*r*=0.23, *P*<.001). Vicarious discrimination related significantly to all outcome variables (depressive symptoms: *r*=0.25, *P*<.001; physical health symptoms: *r*=0.27, *P*<.001; sleep quality: *r*=−0.17, *P*=.003; sleep disturbances: *r*=0.20, *P*<.001).	Focused measures of discrimination that occurred during the pandemic
5. Ermis-Demirtas, et al, 2022 [33]	114 Asian Americans: Chinese (53.5%), Japanese (24.6%), Korean (8.8%), Vietnamese (7%), Thai (3.5%), Filipino (2.6%)	January to May 2021	COVID-19–related discrimination online; COVID-19–related discrimination offline	PHQ-Adolescents, GAD-7	COVID-19–associated discrimination online and COVID-19–associated discrimination in-person variables accounted for an additional 18% variance in the outcome variable (PHQ-Adolescents; *F*_4,109_=60.19; *P*<.001; *R*^2^=.69; adjusted *R*^2^=.68).	It was controlled for childhood trauma.
6. Fanta et al, 2023 [5]	229 East/Southeast Asian parents of a child between 2 and 19 years. Chinese (41%), Taiwanese (27.5%), and Filipino (11.8%).	Not applicable	Discrimination subscale of the General Ethnic Discrimination Scale; COVID-19 Discrimination Fear.	Anxiety and depression subscales of DASS-21^c^	Discrimination predicted higher levels of anxiety (*B*=.38, *P*<.001) and depression (*B*=.23, *P*<.001). Fear of discrimination was positively associated with anxiety (*B*=.18, *P*=.004)	Explored moderating effects of coping styles
7. Haft and Zhou, 2021 [34]	Chinese American college students (134 before and 64 after COVID-19)	Fall semester (September 9, 2019, to December 3, 2019) versus spring semester (February 4, 2020, to March 23, 2020)	Perceived discrimination	Beck Anxiety Inventory; overall media exposure; negative Chinese media exposure	An association between racism and anxiety was found (*r*=0.36, *P*<.001). The COVID-19 pandemic moderated the relationship between racism and anxiety. Negative Chinese media exposure mediated the relationship between racism and anxiety.	Measured during the immediate COVID-19 period
8. Huynh J et al, 2022 [36]	176 Asian American young adults and adolescents from 17 Asian ethnicities, mostly: Chinese (35%), Vietnamese (27%), and Filipinx (18%)	May 2021 to March 2022	Anti-Asian violence: experienced and perceptions of safety	PHQ-9	Three-quarters and two-thirds of participants felt less safe and depressed, respectively. Feeling less safe was more pronounced (*P*<.01) among those who experienced racism and depression.	Youth experience with racism
9. Huynh MP et al, 2024 [6]	3508 Asian Americans: Chinese (19%), Filipino (13.4%), Indian (19.1%), Korean (6.5%), Vietnamese (8.4%), other Asian Americans (15.2%)	January-April 2021	Anti-Asian discrimination; impact of discrimination	PHQ-2, GAD-7	Facing discrimination led to increased odds of psychological distress (OR^d^ 2.10, 95% CI 1.61-2.74).	Examined moderating role of social support, stratified by gender
10. Huynh VW et al, 2022 [35]	380 self-identified East Asian and Southeast Asian American adults: Chinese (26.3%), Japanese (12.6%), Vietnamese (8.7%), Cambodian (8.4%), Korean (7.1%), Filipino (6.1%), Thai (5.5%), Malaysian (4.2%), Taiwanese (3.9%), Indonesian (3.9%), Hmong (3.7%), Laotian (3.7%), Burmese (3.2%), Singaporean (2.6%)	Not applicable	COVID-19–associated discrimination	CES-D^e^, GAD-7	Discrimination was associated with anxiety (*r*=.50, *P*<.01) and depression (*r*=.49, *P*<.01).	Included subdomain of discrimination, and across ethnic identity
11. Ikram et al, 2023 [37]	289 self-identified Asian Indians living in the United States	May 2021 and July 2021	Individual discrimination	Two items: (1) feeling down, depressed, or lonely; and (2) feeling nervous, tense, or worried	Overall, 66% and 46% reported discrimination and poor mental health, respectively. Shapley additive explanations revealed that discrimination is 1 of the 6 predictors of poor mental health.	Use machine learning for data analysis
12. Keum and Choi, 2022 [38]	139 Asian American emerging adults: Chinese (28%), Asian Indian (15%), Filipino (9%), Vietnamese (8%), Japanese (6%), Korean (2%), Thai (2%), Taiwanese (2%), Bangladeshi (1%), Indonesian (1%), Hmong (1%), Laotian (1%), Singaporean (1%), Cambodian (1%), bi/multiethnic (between Asian ethnicities) (9%), others (3%)	June to July 2021	COVID-19 racism	AUDIT^f^, PHQ-9	COVID-19 racism predicted alcohol use severity (standardized effect *β*=.514, 99% bootstrapped CI .314-.713). Furthermore, the mediating analysis showed that racism predicted alcohol use severity through depressive symptoms and drinking to cope motives, accounting for 48% of the variance explained.	Examine mediating effects; small sample size
13. Layug et al, 2022 [39]	1147 adults (aged 18 years or above): White (47.86%), Latinex (11.6%), Black (9.5%), Indigenous (0.96%), mixed (2.88%), Asian Americans and Pacific Islanders (26.68%), Chinese, Japanese, Korean, Filipino, Vietnamese, Indian, Pakistani, Bangladeshi, Sri Lankan, Hmong	March 3 to 15, 2021	Online racial discrimination: individual discrimination, and vicarious discrimination	PHQ-9, GAD-7	Individual online perceived discrimination was a significant positive predictor of STS^g^ (*β*=.52, *P*<.001), depression (*β*=.53, *P*<.001), and anxiety (*β*=.41, *P*<.001). Vicarious online perceived discrimination was positively associated with STS (*β*=.39, *P*<.001), depression (*β*=.39, *P*<.001), and anxiety (*β*=.33, *P*<.001). Asian Americans reported higher vicarious discrimination than Latinx and White Americans.	Explored moderator effect of racial-ethnic identity
14. Lee et al, 2020 [10]	410 Asian Americans		Impact of COVID-19; racial discrimination; social support (Multidimensional Scale of Perceived Social Support)	BAI^h^, CES-D; physical health; sleep health	Asian Americans reported experiencing racism (30%), anxiety (40%), depression (53%), sleep health problems (43%), and physical health problems (15%). Social support had a buffering effect on depression.	Highlighted the buffering effect of social support in the experience of depression
15. Li et al, 2023 [40]	301 Chinese Americans	April 8‐21, 2021	A perceived double threat, online media, and the community COVID-19 racial discrimination	Anxiety (GAD-7)	231 (76.74%) reported threats due to their Chinese ethnic background. Predictors for anxiety were racial discrimination from the local community (OR 0.47, 95% CI 0.39‐0.71, *P*<.001), media/online (OR 0.36, 95% CI 0.26‐0.53, *P*<.001), the perceived threat from the COVID-19 virus (OR 0.33, 95% CI 0.23‐0.51, *P*<.001) and perceived racism threat from Chinese background related to COVID-19 (OR 0.31, 95% CI 0.21‐0.49, *P*<.001).	The perceived double threat was explored
16. Litam et al, 2022	187 Asian Americans		Everyday discrimination Scale (9 items); multigroup ethnic identity; Coping Strategies Inventory-SF	Satisfaction with Life Scale; CES-D revised; BAI	Racism has a negative association with life satisfaction (*b*=−0.253, *P*<.001), and a positive association with depression (*b*=1.479, *P*<.001). Ethnic identity is a positive moderator of the relationship between racism and depression. Coping strategy is a positive moderator of the relationship between depression and life satisfaction.	Highlighted the moderating effect of ethnic identity in racism and life satisfaction association
17. Li et al, 2023 [41]	246 Filipino Americans (who experienced or witnessed COVID-19 racism)	Not indicated	COVID-19–related racial discrimination (modified Everyday Discrimination Scale)	Anxiety (Beck Anxiety Inventory), depression (CESD-R)^i^	COVID-19–related racial discrimination was significantly negatively related to life satisfaction (β=−.208, *P*<.001) and positively related to depression (*β*=.505, *P*<.001) and anxiety (*β*=.496, *P*<.001). Coping strategy moderated the mediated relationship of COVID-19 racial discrimination via depression with life satisfaction.	Focused on those who experienced or witnessed COVID-19 racism
18. Liu Y et al, 2020 [42]	3665 US population	March survey (March 1031, 2020) versus April survey (April 1-28, 2020)	COVID-19–associated discrimination	PHQ-4	Racism increased from March (4%) to April (10%). Non-Hispanic Blacks and Asian Americans experienced increased racism compared to other groups. People who perceived racism reported increased depression (March: OR 0.77; April: OR 1.01)	The total sample included 75% non-Hispanic White
19. Liu T et al, 2022 [43]	565 Asian Americans: Chinese (40%), South Asian (18.1%), Southeast Asian (20.1%), Korean (10.4%), Japanese (4.8%), others (6.6%).	June 2020	COVID-19–specific racism and internalized racism	DASS-21 and PHQ-15	Internalized racism moderated the relations between vicarious racism and psychological distress only for those who were 1.5 generations and above. Individuals who reported higher levels of internalized racism (upper 33%) had higher mean scores of both psychological distress, *F*_2,558_=24.25, *P*<.001, and somatic symptoms, *F*_2,556_=6.86, *P*=.001, when compared to those with low levels of internalized racism (lower 33%). Generational status moderated the relations between vicarious racism and psychological distress (DASS-21) differentially by generation, a test of three-way interactions was significant, *ΔR*^2^=.011, *F*_1,550_=7.53, *P*=.006, *b*=.025, *t*_550_=2.74, *P*=.006.	Examined the complex 3-way interaction among COVID-19–related racism, generation status, and internalized racism
20. Liu MA et al, 2022 [52]	289 Asian Americans	Mid-July 2020	Discrimination experiences (frequency and attribution on race)	Social anxiety (Social Interaction Anxiety Scale), depression (Epidemiological Studies–Depression measure), accumulated stress (Social, Attitudinal, Familial, and Environmental Acculturative Stress Scale–Short Form)	Discrimination was significantly and positively related to depressive symptoms (*B*=7.64, *P*<.001) and alcohol use (*B*=7.05, *P*<.001). This relationship fell short of significance for social anxiety symptoms (*B*=1.55, *P*=.051). About half of the overall sample reported experiencing discrimination (51.6%). Collective self-esteem significantly moderated the relationship between attribution to race and social anxiety (*P*=.021), and internalized racism weakened the relationship between discrimination frequency and depression (*P*=.038).	Moderators were explored.
21. Lu et al, 2022 [44]	218 Asian American college students	March 27 to April 17, 2020	Direct online racial discrimination and general vicarious racial discrimination	GAD-7 and PHQ-9	Overall, 58.7% and 88.1% reported direct online and vicarious racial discrimination, respectively. Direct online racial discrimination was significantly related to depression (*r*=.29, *P*=.003) and anxiety (*r*=.25, *P*<.001). Vicarious racial discrimination also showed a significant relation with depression (*r*=.30, *P*<.001) and anxiety (*r*=.46, *P*<.001).	Examined buffering effect of social support depending on the locus of control factor
22. McGarity-Palmer et al, 2023 [45]	3478 Asian Americans: Chinese (19%), Filipino (13.4%), Indian (18.5%), Korean (6.4%), Vietnamese (8.4%), Pakistani (2.3%), Japanese (3.4%), Cambodian (4.3%), other (5%), multiethnic (2.2%), multiracial (16.8%)	2021	Coronavirus Racial Bias Scale; discrimination	PHQ-4	In total, 24% of Asian Americans (95% CI 21.6-25.6) reported experiencing discrimination. COVID-19–related collective racism was associated with increased psychological distress, above and beyond sociodemographic factors, and other COVID-19–related stressors (*R*^2^=0.36, 95% CI 0.33-0.38).	Performed subgroup analyses
23. Oh S et al, 2022 [46]	270 Korean Americans	Not applicable	Everyday discrimination	BAI and CESD-R	COVID-19–related racial discrimination had significantly positive correlations with depression and anxiety (*R*=.73 and *R*=.61, respectively). Ethnic identity scores were positively correlated with scores of racial discrimination, anxiety, and depression, ranging in magnitude from *r*=.22 to .39.	Explored potential impacts of ethnic identity and coping strategies
24. Oh and Litam, 2022 [46]	725 Asian Americans and Pacific Islanders: Chinese (24.1%), Filipino (23.4%), Korean (17.2%), Vietnamese (7.9%), Japanese (7.0%), Thai (1.9%), other Asian ethnicities (18.3%)	Not applicable	Everyday discrimination	BAI and CESD-R	The path between experiences of racial discrimination and life satisfaction was mediated by anxiety (*b*=−.086, *SE b*=.022, *t*=−3.843, 95% CI −.131 to −.042) and depression (*b*=−.044, *SE B*=.017, *t*=−2.590, 95% CI −.077 to −.011). Coping strategies attenuated the link between discrimination and anxiety, and discrimination and depression.	Examined the role of coping in the relationship between racial discrimination and 2 mediators (anxiety and depression)
25. Pan et al, 2020 [48]	6707 Asian Americans	March 10-31, 2020	COVID-19 stigmatization	PHQ-4	A higher percentage of foreign-born Asian Americans experience racism than US-born Asian Americans (11.2% vs 10.9%). People who experienced COVID-19–related stigmatization reported increased psychological distress (19.9% vs 10.6%). US-born Asian Americans who experienced racism were more likely to exhibit psychological distress than non-Hispanic whites.	Included a larger population and Asian Americans
26. Jun et al, 2021 [49]	254 Asian Americans: Chinese (27.1%), Filipino (13.7%), Korean (10.8%), Vietnamese (10.8%), Japanese (8.7%), other (28.7%)	May 2020	COVID-19 discrimination (occurrence and impact); communication sources about COVID-19	CES-D (20-item)	Both COVID-19 racial discrimination (*b*=4.40, *P*<.001) and previous racial discrimination (*b*=3.05, *P*<.001) were positively associated with depressive symptoms. The negative effects of racism did not vary among different Asian American groups. Not all sources of communication help with depression. Talking with the spouse alleviated depression and interaction on social media depression.	Focused on those 254 who experienced racism
27. Wu et al, 2021 [50]	68,218 data points, tracking 7778 individuals over 13 survey waves	March to September 2020	Acute discrimination	PHQ-4	The mental health gap between Asian Americans and whites (gap=0.98, *P*<.000) is greater than the gap between Asian immigrants and whites (gap=0.18, *P*<.000). 11% of Whites, 22% of Asian Americans, and 21% of Asian immigrants encountered discrimination. A 1-unit within-person increase in acute discrimination leads to a within-person increase in mental disorders by 0.066 units (*P*<.001). Racism mainly explains the disproportionate mental health impact of the pandemic on Asian Americans. US-born Asian Americans experience more racism and anxiety than foreign-born Asian Americans.	Larger population and hone in on Asian American experience from a large dataset
28. Zhou et al, 2023 [51]	Three waves of Asian Pacific Islander students across campuses: Fall 2019 (n=3929), Spring 2020 (n=7813), and Fall 2020 (n=4804).	September-December 2019, March-May 2020, September-December 2020	COVID-19–related discrimination	PHQ-9 and GAD-7	In Fall 2020, experiencing COVID-19–related discrimination was associated with 1.90 greater odds of moderate to severe depression (95% CI 1.13‐3.19; *P*=.016), 2.15 greater odds of severe depression (95% CI 1.29‐3.58; *P*=.003), 1.72 greater odds of moderate to severe anxiety (95% CI 1.07-2.75; *P*=.024), and 1.77 greater odds of severe anxiety (95% CI 1.04‐3.01; *P*=.035).	Large sample inclusive of young adult Asian Pacific Islanders

a GAD-7: General Anxiety Disorder-7.

b PHQ: Patient Health Questionnaire.

c DASS-21: Depression and Anxiety Stress Scales.

d OR: odds ratio.

e CES-D: Center for Epidemiologic Studies Depression Scale.

f AUDIT: Alcohol Use Disorders Identification Test.

g STS: Secondary Traumatic Stress.

h BAI: Beck Anxiety Inventory.

i CESD-R: Center for Epidemiologic Studies Depression Scale Revised.

### Assessed Domains of Racism

As indicated in the previous section, racism encompasses multiple dimensions and forms of expression that depend broadly on social contexts and many other factors. To provide greater insights into this phenomenon as gleaned from our integrative review, a clear understanding of how the authors described various forms of racism is critical to providing a complete picture of what Asian Americans experienced during the COVID-19 pandemic.

Despite the variability in the measurements used in assessing racism among these 9 articles, the victims experienced racist expressions and acts in identifiable ways. Everyday discrimination is defined as the perception of “being treated with less courtesy and respect” [41,42,50,53]. Beyond lack of courtesy and respect, additional descriptors of racism include “receiving poor services at restaurants or stores” [42,50,53,54]. COVID-19–associated acute discrimination was captured by a shorter version of a questionnaire, including only 4 elements of racism (treated with less courtesy and respect, receiving poorer service, being threatened or harassed, and being subjected to others’ fear of COVID-19) [50]. Some victims reported exposure to more aggression, such as physical attacks or verbal attacks of “go back to your own country” [42,50,53,54]. However, some researchers captured more subtle expressions of racism described, for example, “people assume my English is poor due to my race” or “you are called names or insults” [34,55]. Specifically relevant to mental health, the concept of racial discrimination vigilance is worth considering. It is defined as “physical, behavioral, cognitive, and emotional attentiveness to the environment in anticipation of experiencing racism” due to repeated exposure to racism [11]. More subtle expressions of racism experience are described as vicarious racism, which is “indirectly hearing about or seeing racist acts committed against either a member of one’s racial group (friends or family members) personally or in the news” [11].

### Assessed Domains of Mental Health

#### Depression

All 29 articles consistently reported depression as a primary outcome, establishing a direct link between the experience of racism and depression among Asian Americans during the COVID-19 pandemic [5,6,11,31-34,36-39,41-48,50-58]. The association between racism and depression among Asian Americans during the COVID-19 pandemic is profound, as derived from moderate sample size studies and large probability-based, nationally representative samples (Table 2) [50,55]. Most publications reported statistically significant relationships between racism and depression. Consistent with existing literature, Asian American individuals exposed to racist expressions directed at other Asian American groups (vicarious racism) were more likely to experience depression [11,22,31,36,44,48,55]. Being exposed to racism in all forms (online-direct, online-vicarious, in-person–direct, in-person–vicarious, hate-related Sinophobia [defined as fear or dislike of China], and Sinophobia in media) was significantly associated with depression, validating our understanding that social environments of hatred and stereotypes can influence the psyche and mental health of those experiencing racism [22,36,39,58]. Foreign-born Asian Americans and US-born Asian Americans experienced the highest COVID-19 stigma among eight ethnoracial groups: (1) White only, non-Hispanic; (2) White only, Hispanic; (3) Black only; (4) Asian only, foreign born; (5) Asian only, US-born; (6) Asian of mixed race; (7) non-Asian of mixed race; and (8) Indigenous only [53,59,60]. Focusing specifically on racism related to the COVID-19 pandemic, Asian Americans who experienced COVID-19 stigmatization were significantly more likely to report psychological distress (depressive symptoms) than those who did not [53]. COVID-19 stigmatization refers to describing a characteristic or a group of people in a way that shows strong disapproval related to the COVID-19 pandemic; it is a form of discriminatory expression [53]. In a large population-based study evaluating the mental health impact of the COVID-19 pandemic, US-born Asian Americans disproportionately experienced more significant levels of depression than foreign-born Asian Americans, suggesting that demographics and acculturation may explain differing racism experiences [50].

#### Anxiety

Researchers used various tools in multiple domains of racism and found that anti-Asian sentiments have statistically significant associations with anxiety [5,6,11,18,22,31,33-35,38-41,43,44,46,47,51,52,55,56]. Cheah et al 22found that Asian Americans who self-reported more significant vicarious racism had more symptoms of anxiety when controlling for sociodemographic characteristics. The strength of this association was higher than that measured among African Americans during the same assessment time frame around the COVID-19 pandemic, possibly suggesting that this social crisis had a more ethnic-specific impact [9]. Similar to the finding on depression, all types of racial discrimination and Sinophobia were associated with anxiety among adult Asian Americans [22]. In a survey study of 410 Asian American participants who experienced racism during the pandemic, increased incidents of discrimination significantly predicted anxiety symptoms, and social support had a buffering effect for anxiety [44]. Of interest is the damaging impact racism had on younger Asian Americans. Among Asian American youths, anxiety symptoms and internalizing problems were associated with all types of racial discrimination and Sinophobia, suggesting detrimental effects of racism experienced either directly or indirectly through their parents’ experience [22,31,37]. Cheah et al [22] further highlighted that vulnerable youths may be more impacted by COVID-19–related racism because these youths are exposed not only to direct racism but also to the indirect impact of their parents’ victimization experiences, which may translate into increased family stress, a hostile family environment, and negative parenting.

#### Potential Moderators of Depression and Anxiety

Our analysis identified potential moderating factors influencing the relationship between racism and mental health outcomes of depression and anxiety. The synthesis of the data points to the importance of social support in reducing the impact of racism; Asian Americans who experienced a greater incidence of discrimination with less social support reported significantly more depressive symptoms [6,54,55]. In addition, coping strategies and collective self-esteem moderated the relationship between COVID-19 racial discrimination and depression [41,43,46,52,56]. This relationship between racism and anxiety exists through the mediating effect of harmful Chinese media exposure, which is the exposure a person has to the negative portrayal of Chinese immigrants in the media [29,33]. Examples of negative portrayals of Asian Americans may include roles demonstrative of submissiveness, sexual fetish, perpetual foreigners, and model minority. Negative displays or mentions of Asian Americans in media have been shown to incubate and foster racist sentiments against Asian Americans throughout history, particularly during the COVID-19 pandemic, ultimately having harmful effects.

Considering the historical perspective of racism against Asian Americans in the United States, the COVID-19 pandemic intensified the impact of racism on Asian Americans’ mental health, as shown in a moderating effect of the COVID-19 pandemic on the relationship between racism and anxiety [33]. A comparative assessment of social media usage before and after March 16, 2020, indicated that the number of hashtags associated with anti-Asian sentiments (eg, #Chinesevirus) increased significantly. Because of the lockdown mandate, online indirect racism became more common, translating into some challenges for accurately assessing the mental health impact each form of racism may have had on Asian American communities.

## Discussion

### Principal Findings

Our integrative review synthesis of the relationships between COVID-19–related racism and mental health indicates that there are positive correlations between racism, depression, and anxiety. This study represents a comprehensive, up-to-date integrative review of the impact of COVID-19–related racism on mental health outcomes experienced by Asian Americans living in the United States. We identified 2 significant mental health outcomes, including depression and anxiety. Potential factors such as the portrayal of nonstereotypical roles of Asian Americans, effective framing of news media, and social support may mitigate the impact on mental health outcomes to counteract this difficult-to-avoid exposure [29,33]. Researchers described further investigations of moderating factors such as coping style, ethnic identities, and social support to lay the groundwork for potential mitigation [5,6,43,44,56,61]. Strengthening ethnic identity and coping strategies are possible avenues to empower individuals to lessen the impact of racism on mental health.

Our original intent was also to include the impact of racism in the form of physical assaults or crimes perpetrated against Asian Americans during the COVID-19 pandemic. However, we found few that examined direct links between racism and bodily harm. According to the private organization assessment of hate crimes during the COVID-19 pandemic, physical assaults on Asian Americans and Pacific Islanders increased to 1900 cases in 2020 in the United States, coinciding with the pandemic’s peak [62]. Although it is indisputable that hate crimes against Asian Americans dramatically increased during the pandemic, the limited availability of systematically collected data beyond anecdotal reports is possibly due to the lack of a standardized reporting system and the hesitancy of victims to report the crime [63].

While acknowledging that Asian Americans as a group comprise many ethnic origins and languages, this review suggests that Asian Americans experienced the impacts of racism similarly across all ethnic groups. However, it is noteworthy that the experience of racism and its effects on mental health differs between US-born Asian Americans and foreign-born Asian Americans [50,53]. The long-existing view of Asian Americans as “perpetual foreigners” may contribute to the fact that US-born Asians reported a sense of not belonging in either their native countries or the United States. Similarly, the experience of racism differs among generations (first or foreign-born vs second or US-born vs third or US-born) [3,40,43,47].

Although our integrative review revealed that depression and anxiety are significant sequelae of COVID-19–related racism among Asian Americans, some studies identified sleep disturbance as a secondary consequence of racism. Because mental health and sleep are firmly connected, and sleep disturbance can be viewed as a mental health construct (even though it is frequently measured as physiological health), we did not include this outcome as a separate category [58,61,64]. The interconnectedness of these variables emphasizes the overarching influences of racism on mental health that affect both the mind and body.

The impact of COVID-19–related racism on mental health among ethnic minority groups share similarities, yet there are some unique aspects among Asian Americans. Blacks and Latinx Americans also experienced worse depression and anxiety as COVID-19–related racism escalated [11,42,50]. Although all minority groups in the United States have experienced historical and systemic racism, COVID-19–related racism affected Asian Americans or persons who present with Asian phenotypes uniquely because they have been blamed for the origin and the transmission of SARS-CoV-2. Although comparing the deteriorating mental health experienced by Asian Americans and other minority groups is insightful, overemphasis on this difference may prove to be divisive rather than unifying. Further, within Asian American ethnic groups, differences exist in education levels, household incomes, and access to social benefits. Analyzing data as a whole without recognizing these differences may risk ignoring more profound health disparities in some subgroups. Regardless of minority group, a comprehensive approach to addressing racism should include “initiatives to raise awareness levels of the pervasiveness of inequities in health, build empathy and support for addressing inequities, enhance the capacity of individuals and communities to actively participate in intervention efforts and implement large scale efforts to reduce racial prejudice, ideologies, and stereotypes in the larger culture that undergird policy preferences that initiate and sustain inequities” [65]. All 29 articles reviewed here utilized one type of research design, cross-sectional survey study designs, and were conducted during the COVID-19 pandemic in March 2020. Most studies in our review used a small sample size, making it difficult to generalize to the larger population. Although we intended to include all Asian American groups in our original search, most studies included Chinese Americans, and some studies included participants from a single ethnicity, such as Korean, Filipino, and Asian Indians [22,31,34,37,40,41,47,56]. Since we included only studies conducted in the United States, we should avoid generalizing study findings in different geographical and cultural contexts outside the United States. Importantly, Asian Americans are nonmonolithic, with diverse socioeconomic and educational backgrounds, and it is challenging to describe precisely how racism affects each group [29].

### Clinical Implications and Future Research

#### Overview

The act of racism (with its plethora of presentations) has a range of impacts on the victim that span from mental health deterioration, discriminatory effects (employment), and physical crime (physical assault). Our integrative review results address an essential query by summarizing how racist sentiments impact mental health and well-being among Asian Americans living in the United States during the COVID-19 pandemic. This scientific knowledge provides a foundation for further research directions and clinical implications addressing this urgent issue with the following recommendations.

#### Public Awareness and Individual-Level Education

Following the US Commission on Civil Rights 2023, Statutory Enforcement Report on the Federal Response to Anti-Asian Racism in the United States, there is a need to increase public awareness campaigns and education about all dimensions of racism, systemic racism, the prevalence of racism, and anti-racism initiatives [59]. Given the profound mental health impact of racism, a culturally tailored educational program should be provided to Asian Americans, their family members, and the community. Academic programs may include a clear understanding of the legal right to safety and security, with attention to social justice and equity. These public awareness campaigns and education efforts should positively impact personal and systemic changes with measurable and meaningful outcomes. In raising public awareness about anti-Asian sentiments in America, it is essential to include all types of racism. Evidence shows that subtle displays of racism are more prevalent than the overt type yet they can still potentially be harmful. Biases, left unrecognized and not dealt with, have the potential to fuse into systemic racism. The success of public awareness and education depends on financial support and advocacy for funding to build and sustain these essential initiatives and address the root causes of these acts and words.

#### Programs to Document Incidents of Racism and Hate Crimes

There is an urgent need to establish programs to empower Asian American community members to recognize racism and report all occurrences of racism. Ponce (2022) indicates that only 6.9% of Asian Americans reported race-based hate, and 62.4% of these reported no exposure to race-based unfair treatment [63]. This report revealed that there is discordance between reporting and actual exposure; Asian Americans were found to experience high psychological distress, forgo necessary medical care, and have personal conflicts and insecurity about safety [62]. Since racist incidents often occur among vulnerable Asian Americans (eg, females, older adults, and young victims), the provision of specific “how-to” tools for recognizing the problem and promptly filing reports is of importance. To create action plans to address racism in the United States, accurate and timely documentation of the incidents is necessary [29,49]. As announced by the Biden administration, a system for reporting hate crimes against Asian Americans was established and funded. However, a barrier to this effort may be a lack of public awareness and education programs to educate vulnerable victims about recognizing and reporting these crimes.

#### Intervention Programs for Mental Health

Our findings suggest a critical need to design interventions such as culturally responsive trauma-informed treatment to address the mental health needs of victimized Asian Americans. Culturally responsive, trauma-informed intervention includes the recognition that the current anti-Asian sentiment may activate intergenerational trauma, reinforce cultural mistrust, and echo centuries of historical oppression [41]. Counseling approaches and intervention programs aiming to address this traumatic psychological distress within the context of COVID-19–related racism should incorporate relevant contributing factors that may mediate or moderate the effects of racism on mental distress [54]. Culturally sensitive trauma-informed care refers to the capacity for health care professionals to provide trauma-informed assessment and intervention that acknowledges, respects, and integrates patients' and families' cultural values, beliefs, and practices.

We need to advocate for resources supporting intervention programs to address mental health that are culturally relevant and anchored in engaging family- and community-level initiatives [12,66-68]. In exploring the type of social support that may help alleviate mental health (communication method about the COVID-19 pandemic), not all communication sources help alleviate depression among Asian Americans who experienced racism [54]. Talking with a partner/spouse was shown to be the type of communication with the most positive impact on depression, suggesting family- or community-level intervention programs to be the most helpful [54]. More research is needed to understand the moderating impact of social support and ethnic identity as a possible intervention for those who experience racism-related depression.

### Conclusions

This integrative review summarizes the gravity of the mental health outcomes of depression and anxiety among Asian Americans associated with a wide range of racism related to the COVID-19 pandemic. Our findings suggest a critical need to design interventions such as culturally responsive trauma-informed treatment to address various Asian American groups’ urgent mental health needs. Furthermore, more research is needed to examine the long-term impact of discrimination on mental health in Asian Americans and address the ongoing health inequity in health care practice.

## Supplementary material

10.2196/63769 Checklist 1 PRISMA checklist.

## Abbreviations

JBI: Joanna Briggs Institute
OR: odds ratio
PRISMA: Preferred Reporting Items for Systematic Reviews and Meta-Analyses
